# Supplementary material for: GtTR: Bayesian estimation of absolute tandem repeat copy number using sequence capture and high throughput sequencing
Source: BMC Bioinformatics. 2018 Jul 16;19:267. doi: 10.1186/s12859-018-2282-3 (PMC6048696; doi:10.1186/s12859-018-2282-3)
Supplement: Supplementary file 2 — Supplementary methods. Includes supplementary information on analysis and supplementary Figures S1–S13. (DOCX 1956 kb) [file 12859_2018_2282_MOESM2_ESM.docx]

**Supplementary Methods**

**VNTRtyper:** We developed VNTRTyper, an in-house tool to genotype TRs from long read PacBio sequencing data (manuscript in preparation). Long read sequencing data often have high error rate due to the inherent limitation of these technologies, which can affect the accuracy of VNTR typing. To circumvent this, VNTRTyper firstly identifies reads that span the region and uses a profile HMM based approach to count the multiplicity of repeat unit in a read.

VNTRtyper uses a profile HMM to model the alignment of long reads to a tandem repeat and its flanking regions. Briefly, the model uses a probabilistic finite automaton to present matched states and the cost of transition between a state to another (Supplementary Figure 1). The parameters for the profile HMM (the costs associated to transitions) are determined by mutation rates among repeat copies, as well as the error profile of the sequencing technology. It then aligns long reads to the model by applying a dynamic programming algorithm to find the best path (path with the minimal cost) to generate the read sequence. Figure 1 depicts the different states during the alignment of the repeat unit. For example, a read (R) which spans through the lflank and rflank (flanking sequences) will enter the alignment start (S) state and if the sequence matches the reference sequence, the path through the model will pass from S state to position (P) state. If the sequence contains a deletion relative to the reference, the path will go through the delete (D) state before transitioning to the next P state; if the sequence contains an insertion relative to the reference, the path will go through the insert (I) state before transitioning to the next P state. This cycle is repeated until the whole read R is aligned and reaches the end (E) state.

Supplementary Figure 1: Different states of profile HMM

VNTRtyper can be accessed from <https://github.com/mdcao/japsa> and it can be deployed using script name jsa.tr.longreads.

Usage: jsa.tr.longreads [options]

Options:

--reference=s Name of the reference genome

(REQUIRED)

--bamFile=s Name of the bam file

(REQUIRED)

--output=s Name of the output file, - for stdout

(default='-')

--xafFile=s Name of the regions file in xaf

(REQUIRED)

--flanking=i Size of the flanking regions

(default='30')

--qual=i Minimum quality

(default='0')

--iteration=i Number of iteration

(default='1')

--nploidy=i The ploidy of the genome 1 = happloid, 2 = diploid. Currenly only support up to 2-ploidy

(default='2')

--prefix=s Prefix of temporary files, if not specified, will be automatically generated

(default='')

--help Display this usage and exit

(default='false')

Example of the usage:

$ jsa.tr.longreads --reference hg19.fas --bam PacBio.bam --flank 30 --xaf VNTR.xaf --output output/PacBio_VNTR.results

Input files for jsa.tr.longreads includes reference genome (fasta format), an aligned bam file and a XAF file which includes the information on the repeats (Target ID, chromosome, start position, end position, size of the region, period, repeat unit, left flank size, right flank size, target region including the flanking and the sequence of the repeat unit). The output from jsa.tr.longreads provides an alignment of each repeat unit and the flanking sequences for each sequence read which spans the region and provides an estimation of the genotype supported by each read. A customized awk script was used to obtain the genotype information from the output file.

$ awk '$1~"^##" && $1!="##reference" && NR > 3 {if($5<=1.80) {print $2}}' <result.file> | sort | uniq -c

The output of the awk script contains the number of reads observed for each genotype (i.e. number of repeat unit). Genotype estimates were only considered if there was at least two supporting reads per genotype. These genotype estimates per target region were used for the GtTR analysis.

**GtTR:** GtTR analysis script can be accessed from <https://github.com/mdcao/japsa> and can be deployed using script name jsa.dev.captureVNTR.

Usage: jsa.dev.captureVNTR [options]

Options:

--xafFile=s Name of repeat file

(default='VNTR.xaf')

--reference=s Name of reference genome

(default='hg19.fas')

--target=s Where to write the target

(default='target.fa')

--technology=s Technology: pacbio or illumina

(default='pacbio')

--directory=s Directory with depth files

(default='./')

--stat=i 0,1,2

(default='2')

--readLength=i Read length

(default='250')

--stage=i Stage of processing:

0: Generate hmm profile, technology parameter is required

1: Extract target sequences, prepare for alignment

2: Look for reads spanning any of the repeats

3: Read depth information extraction

4: ---

5: Read depth analysis

6: Read depth analysis with likelihood

(default='6')

--output=s Name of output file, - for standard out

(default='-')

--pad=i Gaps

(default='10')

--resample=s reference sample

(default='null')

--resAllele=s reference alleles

(default='null')

--HPD=s High Posterior Density interval between 0 and 100

(default='95')

--help Display this usage and exit

(default='false')

Example of the usage:

$ jsa.dev.captureVNTR --xaf VNTR.xaf –dir /PATH/DIRECTORY/ –stage 6 --output Results.dat --resample ReferenceReaddepth.rdepth –resAllele PacBio_refAllele.txt --HPD 90 Directory_Rdepth/

Input files for jsa.dev.captureVNTR includes a XAF file which includes the information on the repeats, genotypes of the target in the reference sample (an average of the 2 alleles), the read depth of the reference sample and the read depth files of the test samples. Read depth files can be obtained using the jsa.tr.trdepth script described below. The output from jsa.dev.captureVNTR would contain the genotype estimate, HPD interval range of the genotype estimate, HPD interval value, and standard error.

Read depth calculation for GtTR analysis were performed using an in-house tool, which can be accessed from <https://github.com/mdcao/japsa> and it can be deployed using script name jsa.tr.trdepth.

Usage: jsa.tr.trdepth [options] bamFile1 bamFile2 bamFileN

Options:

--xafFile=s XAF file containing repeat information

--qual=i Minimum mapping quality

(default='0')

--depth Include depth coverage (R3 and S3)

(default='false')

--filterBits=i Filter reads based on flag. Common values:

0 no filter

256 exclude secondary alignment

1024 exclude PCR/optical duplicates

2048 exclude supplementary alignments

(default='0')

--output=s Name of output file, - for standard out

(default='-')

--maxFragment=i Fragment size, 0 if single end

(default='0')

--readLength=i Read length

(default='250')

--help Display this usage and exit

(default='false')

Example of the usage:

$ jsa.tr.trdepth -x VNTR.xaf -q 20 –o output/Test.rdepth Bamfile1.bam Bamfile2.bam Bamfile3.bam

Input files for the jsa.tr.trdepth includes a XAF file which includes the information on the repeats (Target ID, chromosome, start position, end position, size of the region, period, repeat unit, left flank size, right flank size, target region including the flanking and the sequence of the repeat unit) and aligned bam files. The output from jsa.tr.trdepth provides read depth information for each target.

**Capillary Electrophoresis Results of the PCR sizing analysis**


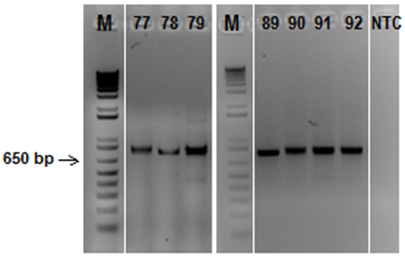


**Supplementary Figure 2:** Electrophoretic profile of VNTR_8 (size - hg19: 502bp) in the 7 family members in study, in a 1.5% agarose gel (M – 1kb plus DNA ladder, 77 – NA12877, 78 – NA12878, 79 – NA12879, 89 –NA12889, 90 – NA12890, 91- NA12891, 92 – NA12892, NTC – no template control)

| **NA12877** | **NA12890** |
| --- | --- |
|  |  |
| **NA12878** | **NA12891** |
|  |  |
| **NA12879** | **NA12892** |
|  |  |
| **NA12889** | **NTC** |
|  |  |

**Supplementary Figure 3:** Capillary electrophoresis plots of VNTR_8 (size - hg19: 502bp) in the 7 family members in study.


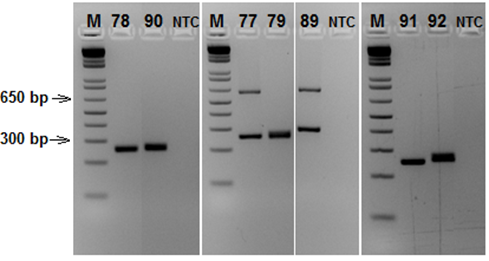


| **NA12877** | **NA12890** |
| --- | --- |
|  |  |
| **NA12878** | **NA12891** |
|  |  |
| **NA12879** | **NA12892** |
| 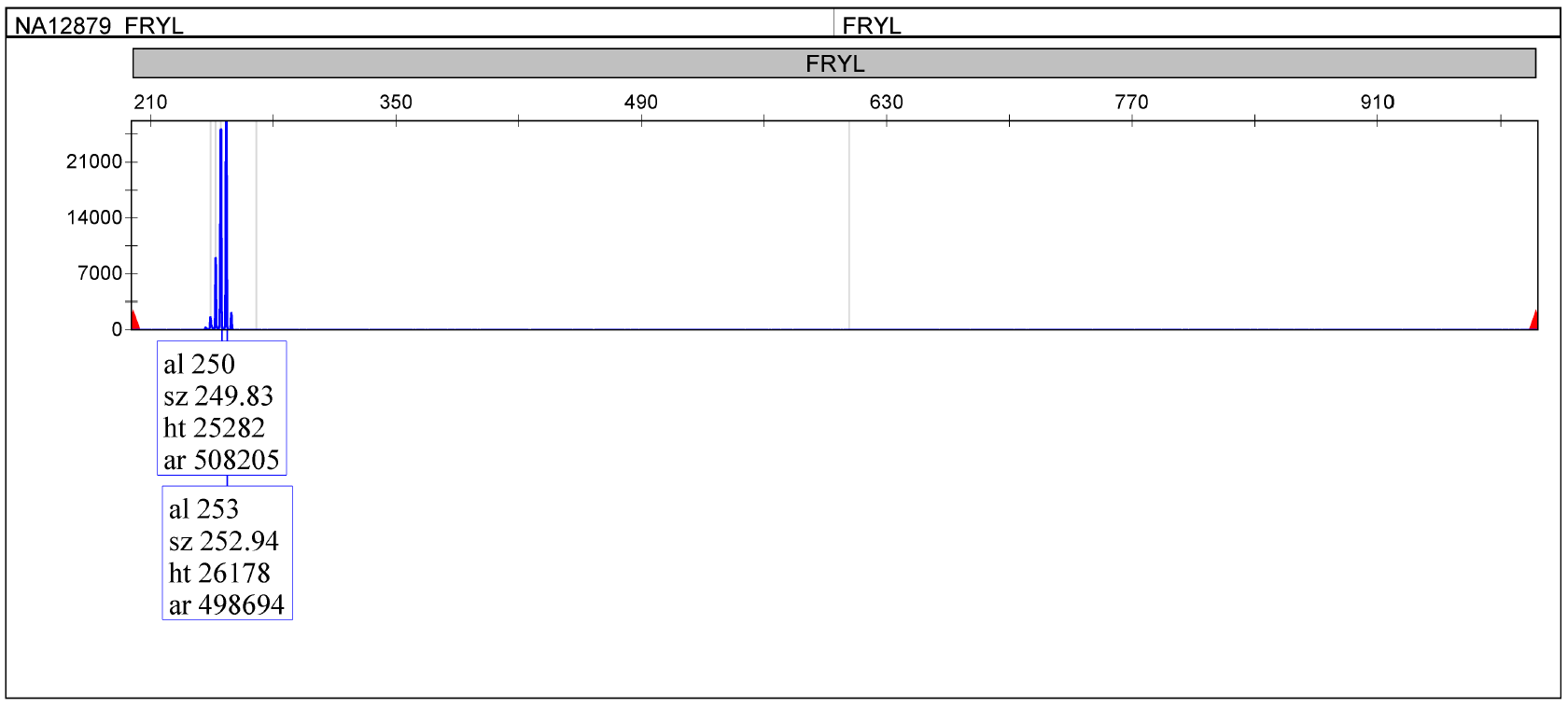 |  |
| **NA12889** | **NTC** |
|  |  |

**Supplementary Figure 4:** Electrophoretic profile of VNTR_32 (size - hg19: 594bp) in the 7 family members in study, in a 3% agarose gel (M – 1kb plus DNA ladder, 77 – NA12877, 78 – NA12878, 79 – NA12879, 89 –NA12889, 90 – NA12890, 91- NA12891, 92 – NA12892, NTC – no template control)

**Supplementary Figure 5:** Capillary electrophoresis plots of VNTR_32 (size - hg19: 594bp) in the 7 family members in study.

| **NA12877** | **NA12890** |
| --- | --- |
| 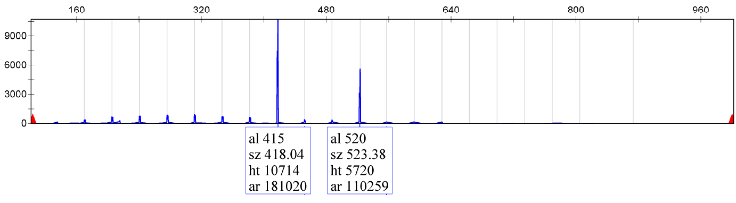 | 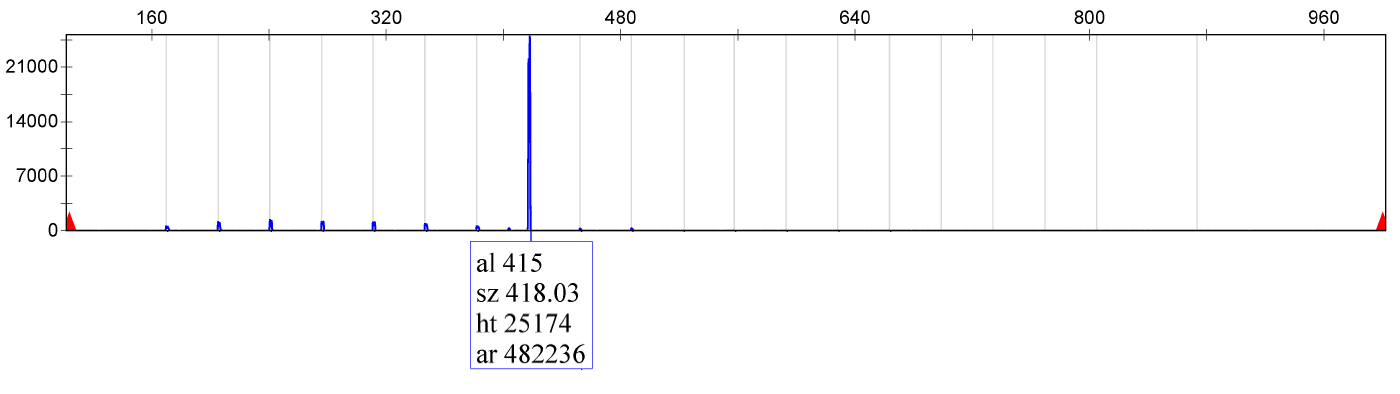 |
| **NA12878** | **NA12891** |
| 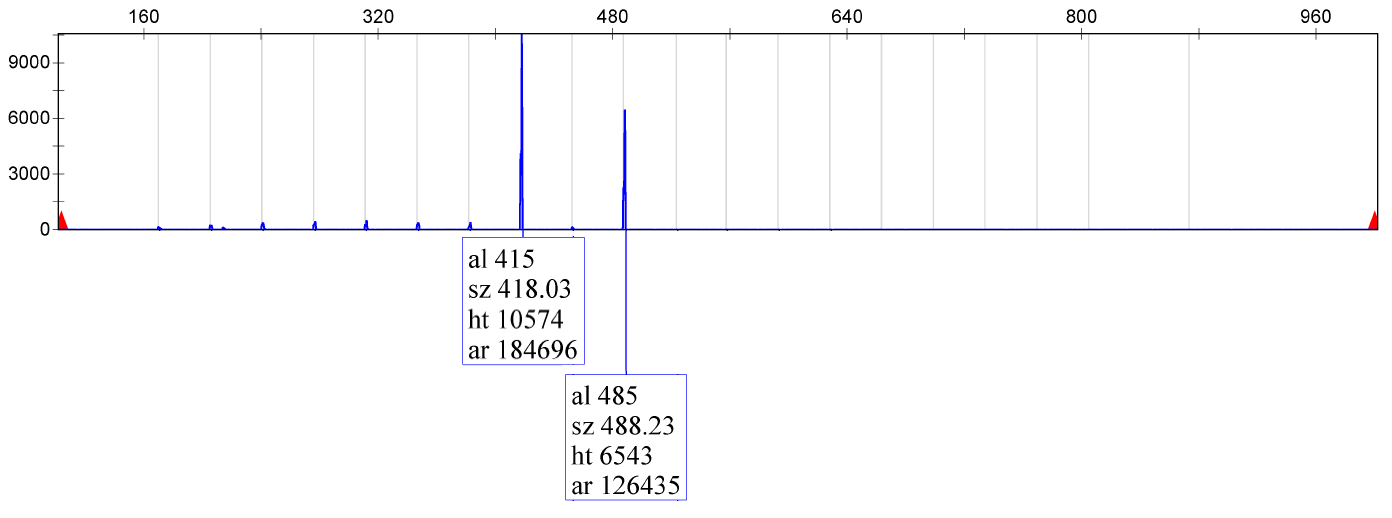 | 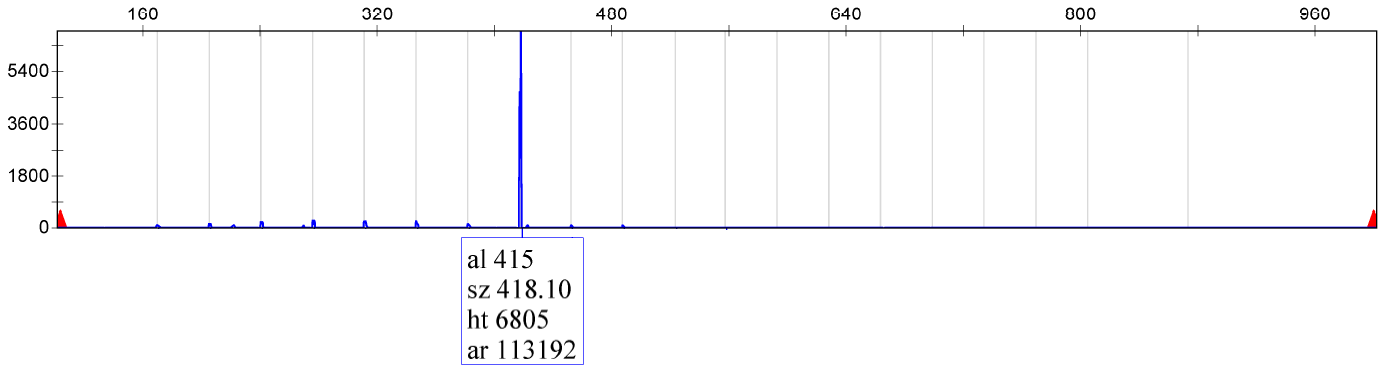 |
| **NA12879** | **NA12892** |
| 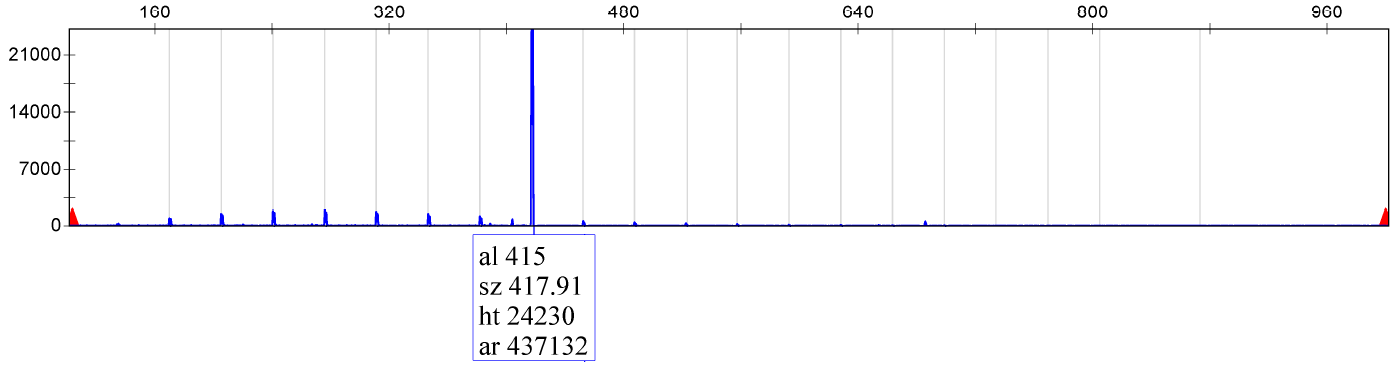 | 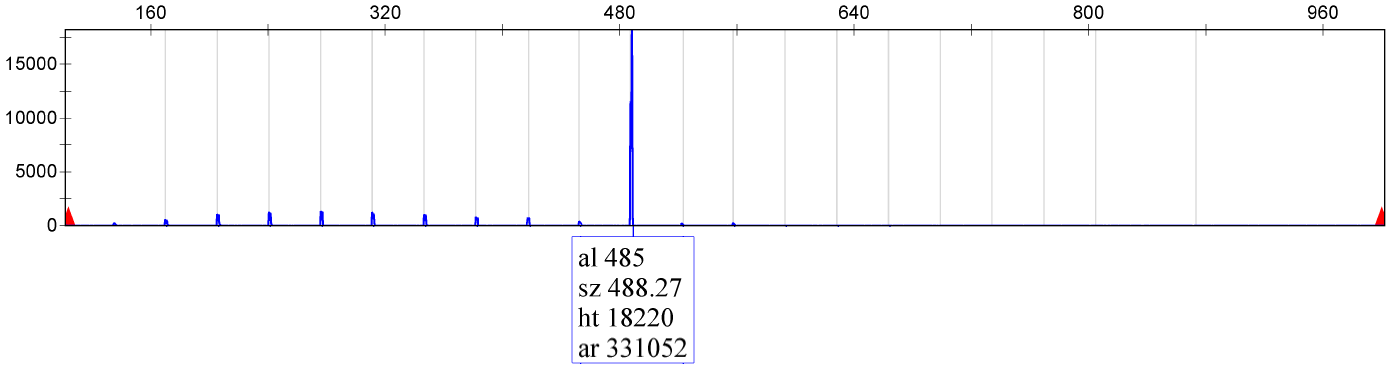 |
| **NA12889** | **NTC** |
| 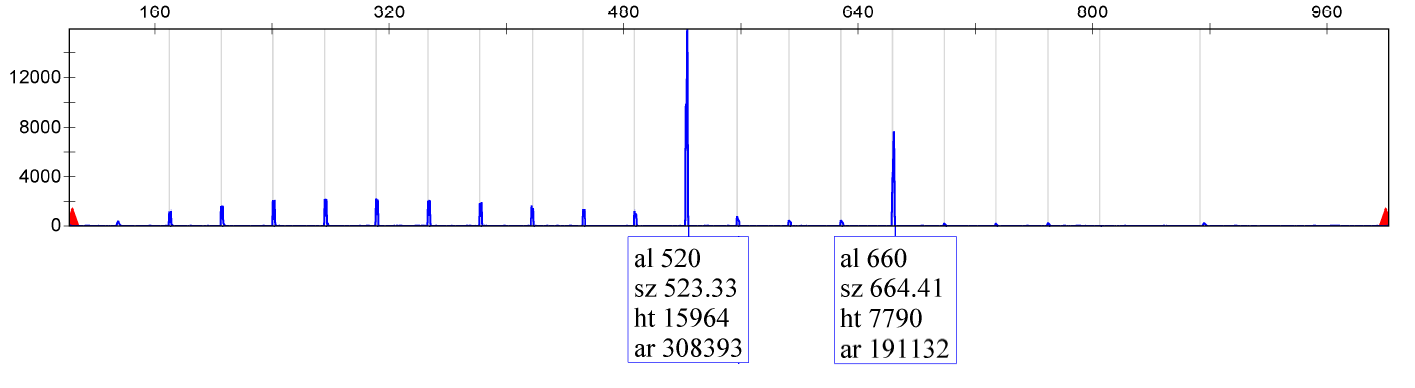 |  |

**Supplementary Figure 6:** Capillary electrophoresis plots of VNTR_57 (size - hg19: 544bp) in the 7 family members in study.

| **NA12877** | **NA12890** |
| --- | --- |
|  |  |
| **NA12878** | **NA12891** |
|  |  |
| **NA12879** | **NA12892** |
|  |  |
| **NA12889** | **NTC** |
|  |  |

**Supplementary Figure 7:** Capillary electrophoresis plots of VNTR_86 (size - hg19: 245bp) in the 7 family members in study.

| **NA12877** | **NA12890** |
| --- | --- |
|  |  |
| **NA12878** | **NA12891** |
|  |  |
| **NA12879** | **NA12892** |
|  |  |
| **NA12889** | **NTC** |
|  |  |

**Supplementary Figure 8:** Capillary electrophoresis plots of VNTR_87 (size - hg19: 685bp) in the 7 family members in study.

| **NA12877** | **NA12890** |
| --- | --- |
|  |  |
| **NA12878** | **NA12891** |
|  |  |
| **NA12879** | **NA12892** |
|  |  |
| **NA12889** | **NTC** |
|  |  |

**Supplementary Figure 9:** Capillary electrophoresis plots of VNTR_93 (size - hg19: 267bp) in the 7 family members in study.

| **NA12877** | **NA12890** |
| --- | --- |
| 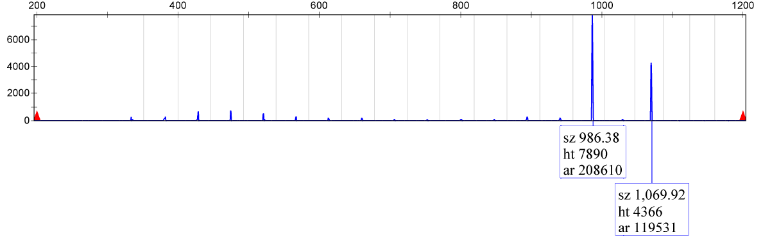 | 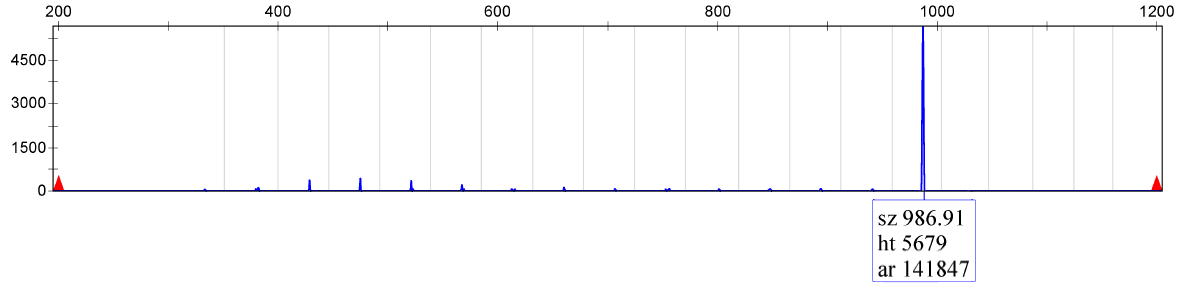 |
| **NA12878** | **NA12891** |
| 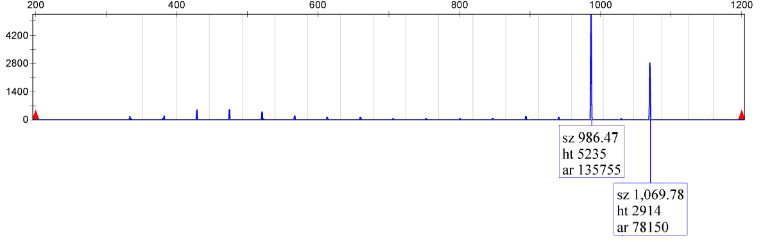 | 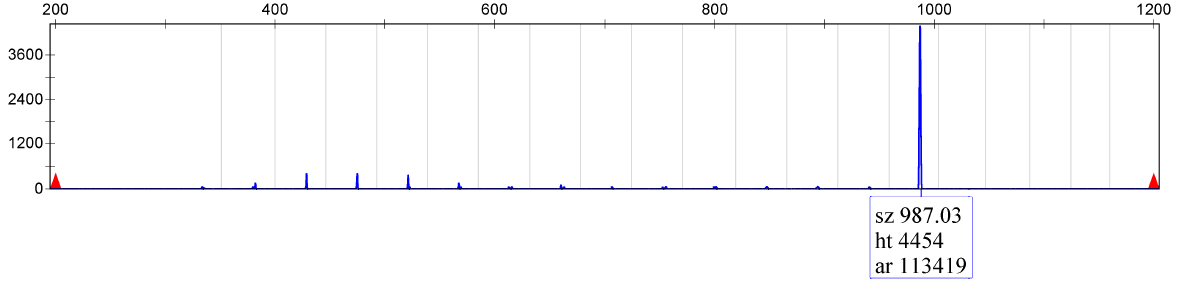 |
| **NA12879** | **NA12892** |
| 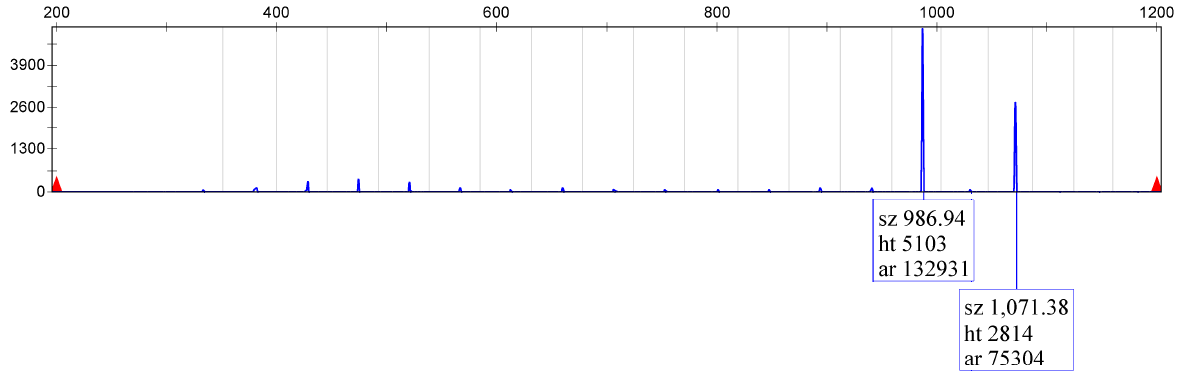 | 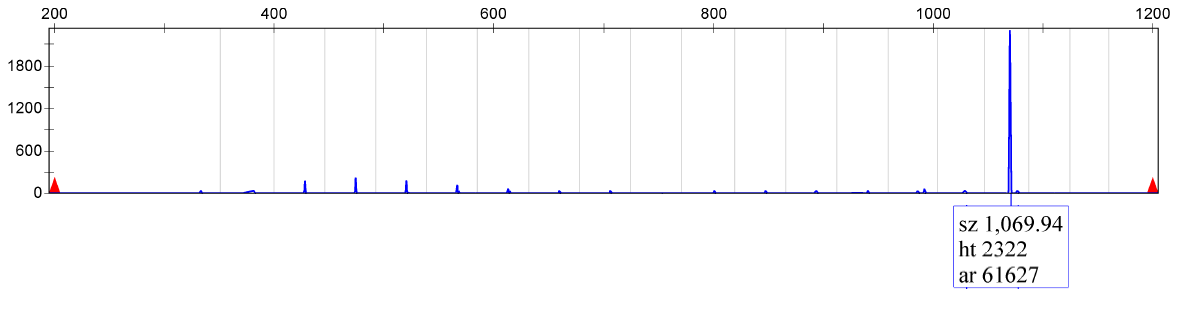 |
| **NA12889** | **NTC** |
| 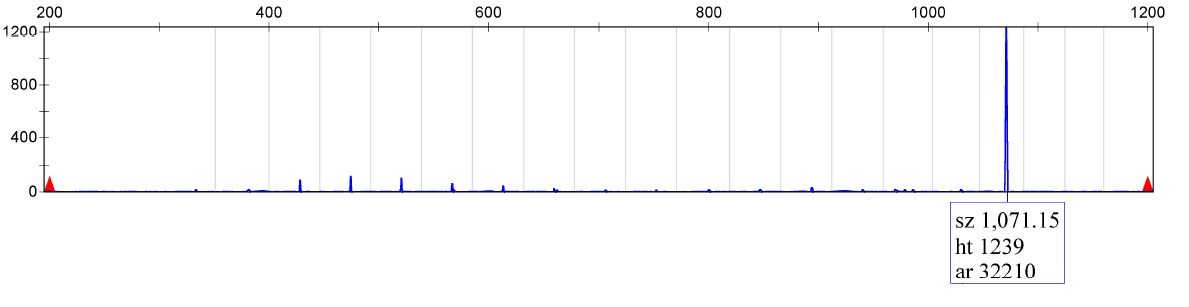 |  |

**Supplementary Figure 10:** Capillary electrophoresis plots of VNTR_109 (size - hg19: 724bp) in the 7 family members in study.

| **NA12877** | **NA12890** |
| --- | --- |
|  |  |
| **NA12878** | **NA12891** |
|  |  |
| **NA12879** | **NA12892** |
|  |  |
| **NA12889** | **NTC** |
|  |  |

**Supplementary Figure 11:** Capillary electrophoresis plots of VNTR_112 (size - hg19: 300bp) in the 7 family members in study.

| **NA12877** | **NA12890** |
| --- | --- |
|  |  |
| **NA12878** | **NA12891** |
|  |  |
| **NA12879** | **NA12892** |
|  |  |
| **NA12889** | **NTC** |
|  |  |

**Supplementary Figure 12:** Capillary electrophoresis plots of VNTR_120 (size - hg19: 112bp) in the 7 family members in study.

**Supplementary Figure 13:** Overall cumulative distribution of half relative width of 95% HPD interval in genotype estimates in targeted capture sequencing data at varying sequence coverage (by down-sampling analysis).
